# Association Between Cholinesterase Inhibitors and New-Onset Heart Failure in Patients With Alzheimer's Disease: A Nationwide Propensity Score Matching Study

**DOI:** 10.3389/fcvm.2022.831730

**Published:** 2022-03-16

**Authors:** Ming-Jer Hsieh, Dong-Yi Chen, Cheng-Hung Lee, Chia-Ling Wu, Ying-Jen Chen, Yu-Tung Huang, Shang-Hung Chang

**Affiliations:** ^1^Division of Cardiology, Department of Internal Medicine, Chang Gung Memorial Hospital, Taipei, Taiwan; ^2^College of Medicine, Chang Gung University, Taoyuan, Taiwan; ^3^Center for Big Data Analytics and Statistics, Chang Gung Memorial Hospital, Taipei, Taiwan; ^4^Division of Geriatrics and General Internal Medicine, Department of Internal Medicine, Chang Gung Memorial Hospital, Taoyuan, Taiwan

**Keywords:** Alzheimer's disease, cholinesterase inhibitors, new-onset heart failure, primary prevention, propensity score matching

## Abstract

**Background:**

Autonomic nervous dysfunction is a shared clinical feature in Alzheimer's disease (AD) and heart failure (HF). Cholinesterase inhibitors (ChEIs) are widely used autonomic modulators in patients with AD, but their primary preventive benefit on new-onset HF is still uncertain.

**Objective:**

This study examined whether ChEIs have a primary preventive effect on new-onset HF in patients with AD.

**Methods:**

This propensity score matching (PSM) study was conducted using data from the National Health Insurance Research Database of Taiwan for 1995 to 2017. Certificated patients with AD and without a history of HF were divided into ChEI (donepezil, rivastigmine, or galantamine) users or nonusers. The primary endpoint was new-onset HF, and the secondary endpoints were myocardial infarction and cardiovascular death after 10-year follow-up.

**Results:**

After screening 16,042 patients, 7,411 patients were enrolled, of whom 668 were ChEI users and 1,336 were nonusers after 1:2 PSM. Compared with nonusers, ChEI users exhibited a significantly lower incidence of new-onset HF (HR 0.48; 95% CI 0.34–0.68, p < 0.001) and cardiovascular death (HR 0.55; 95% CI 0.37–0.82, p = 0.003) but not of myocardial infarction (HR 1.09; 95% CI 0.52–1.62, p = 0.821) after 10-year follow-up. The preventive benefit of ChEI use compared with Non-use (controls) was consistent across all exploratory subgroups without statistically significant treatment-by-subgroup interactions.

**Conclusions:**

Prescription of ChEIs may provide a preventive benefit associated with lower incidence of new-onset HF in patients with AD after 10-year follow-up.

## Introduction

With longer human life spans, the incidences of age-related diseases such as Alzheimer's disease (AD) and heart failure (HF) are increasing (1). Autonomic nervous dysfunction with sympathetic activation and cholinergic neurotransmission deficiency are the shared features of AD and HF (2–4). In healthy people, the parasympathetic system is more dominant than sympathetic tone under resting conditions. However, under stressful conditions, the sympathetic system is activated, and the parasympathetic system is suppressed to help the body respond to the emergency; additional energy expenditure is involved in this. During aging and the development of AD, parasympathetic vagal tone diminishes, and sympathetic tone becomes more predominant; therefore, the incidences of cardiovascular disorders such as hypertension and HF increase in AD (5, 6). Inhibition of sympathetic activity by beta-blockers has been the standard therapy for HF, but the primary preventive effect on the development of HF in the early stages is controversial (7). How parasympathetic activation is involved in HF prevention and treatment is still uncertain and under investigation (8–10).

Cholinesterase catalyzes acetylcholine into choline and acetic acid, and choline is then recycled to make acetylcholine in a continuous process during neuronal synaptic transmission. Cholinesterase inhibitors (ChEIs), such as donepezil, rivastigmine, and galantamine, increase the concentration of acetylcholine through acetylcholine catalysation reduction and have been shown to improve cognitive function significantly; they are widely used in patients with AD (11–13). The effect of ChEIs on cholinergic activation, however, is not limited to the central neurons; the intrinsic cardiac neurons, which regulate the chronotropic and dromotropic functions of the heart, are also affected by ChEIs (14). Therefore, when patients with AD are prescribed ChEIs, clinical manifestations such as bradycardia or sinoatrial or atrioventricular blocks should be monitored (15). In addition to these clinical warning signs, anti-inflammatory and negative chronotropic effects mean that ChEIs may offer potential cardioprotective effects that mainly result in reduced incidence of myocardial infarction (MI) and mortality (16, 17). Regarding effect of ChEIs on caridac muscle, reduction of cardial remodeling and ischemic reperfusion injury have been reported in animal studies, but whether a clinical benefit of ChEIs on the prevention of HF exists remains unclear (18, 19). The aim of this study was to investigate the primary preventive effect of ChEIs on new-onset HF development in patients with AD.

## Materials and Methods

The Institutional Review Board of Chang Gung Memorial Hospital approved the protocol for this cohort study and waived the need for informed consent because all patient data were deidentified before analysis (IRB No. 202100758B1). This study follows the Strengthening the Reporting of Observational Studies in Epidemiology (STROBE) reporting guidelines.

### Data Source

We conducted this national database cohort study of patients with dementia or AD aged ≥40 years by using data from the Taiwan National Health Insurance program, which has been implemented since 1995 and covers nearly 99.9% of Taiwan's population of 23 million. We used claims data to collect demographic information, diagnoses based on the International Classification of Diseases, Ninth Revision, Clinical Modification (ICD-9-CM), treatment procedures, and prescription records. The claims data are characterized by continual longitudinal recording and comprehensive coverage; updated data are released annually by the NHRI. The database information is anonymously collected and protected by a unique identification number for each individual.

### Study Population

**Figure 1:**
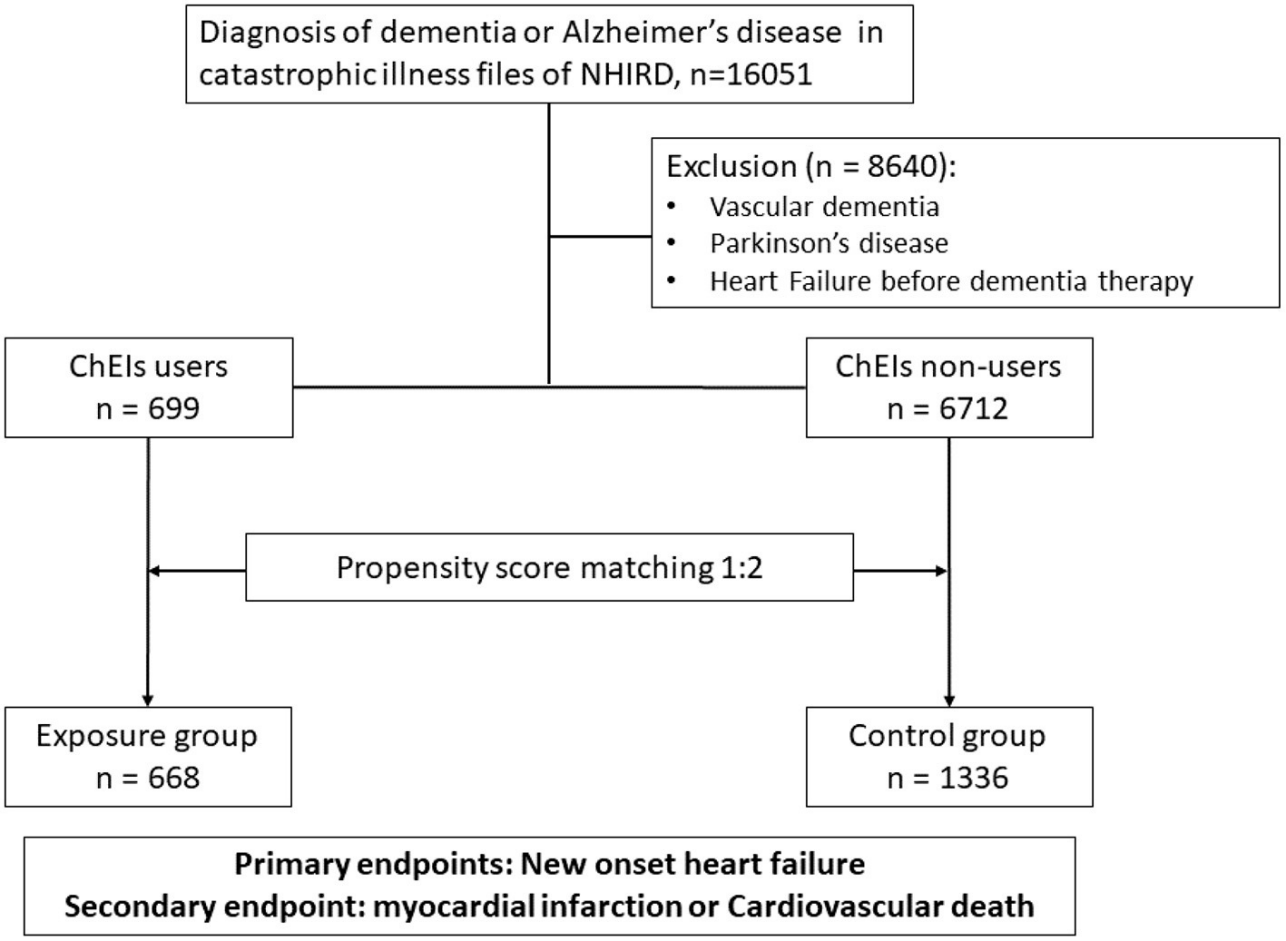
Flow chart of patient enrolment in this study.

Patients aged 40 years or older and newly diagnosed as having dementia or AD (ICD-9-CM codes 290.0–290.9 and 331.0) were identified from the catastrophic illness file of the National Health Insurance Research Database (NHIRD) between 1 May 1995 and 30 September 2017. To reduce the confounding bias of underlying diseases, patients who had a concomitant diagnosis of vascular dementia (ICD-9-CM code 290.4), Parkinson's disease (ICD-9-CM code 332), or occurrence of HF (ICD-9-CM codes 428, 425.4, 425.5, 425.6, 425.7, 425.8, 425.9, 402.01, 402.11, 402.91, 404.01, 404.03, 404.11, 404.13, 404.91, 404.93, 785.51) before dementia diagnosis or treatment were excluded. After exclusion, the remaining patients with AD were divided into either the exposure group (ChEI users) or the control group (ChEI nonusers). The exposure of interest—the use of ChEIs or not—was identified through prescription records. ChEIs included donepezil (Anatomical Therapeutic Chemical Classification System [ATC] code: N06DA02), rivastigmine (ATC code: N06DA03), and galantamine (ATC code: N06DA04). Comorbidities at baseline were identified using ICD-9-CM diagnostic codes at the index date and 1 year before the index date. The ICD-9-CM codes used to identify the study covariates and outcomes are summarized in the supplement. In addition, cardiovascular medications, including antiplatelet and anticoagulation medications, statins, beta-blockers, angiotensin-converting enzyme inhibitors (ACEIs), or angiotensin receptor blockers (ARBs) were also documented. The propensity score matching (PSM) method was employed to balance these baseline comorbidities and use of cardiovascular medications. A flowchart of the study cohort enrolment process is shown in Figure 1.

The index date of initial follow-up was the date of first administration of ChEIs in the exposure group. In the control group, the index date was the date of receiving a diagnosis of AD. The primary outcome was new onset of HF. The definition of new-onset HF in this present study was newly diagnosis of HF at outpatient and/or discharge from hospital (ICD-9-CM codes 428, 425.4, 425.5, 425.6, 425.7, 425.8, 425.9, 402.01, 402.11, 402.91, 404.01, 404.03, 404.11, 404.13, 404.91, 404.93, 785.51). The secondary endpoints were the occurrence of either MI or cardiovascular death. Cardiovascular death was defined as mortality due to cardiovascular etiology (ICD-9-CM codes 390–459). All study participants were followed up from the index date until the occurrence of a relevant event, withdrawal from the insurance program, follow-up at 10 years, or follow-up until 31 December 2018, whichever occurred first.

### Statistical Analysis of Cohort Study

We assessed the differences in baseline characteristics, comorbidities, and medications between the treatment and control groups. Covariates introduced to construct the propensity score included age, sex, comorbidities, and medications. The Cox proportional hazards model and competing risk regression analysis were used to estimate the treatment effects on clinical outcomes. A two-sided *p*-value of < 0.05 indicated statistical significance. All data analyses were conducted using SAS version 9.4 (SAS Institute, Cary, NC, USA).

A subgroup analysis was conducted to determine whether the hazard ratios (HRs) of outcomes for the ChEIs and controls were similar in the prespecified subgroups. The subgrouping factors included age (75 years old); sex; presence of diabetes mellitus, hypertension, or hyperlipidaemia; prior stroke; presence of chronic kidney disease, chronic lung disease, or peripheral vascular disease.

## Results

### Clinical Characteristics of Patients Before and After PSM

From 1 May 1995 to 30 September 2017, a total of 16,051 patients were diagnosed as having AD in the catastrophic illness file of the NHIRD. After excluding 8,640 patients with vascular dementia, Parkinson's disease, or a diagnosis of HF before using ChEIs, a total of 7,411 patients were enrolled in the study. Of the total, 699 participants comprised the exposure group and were treated with ChEIs, and 6,712 comprised the control group and were patients undergoing conventional therapy. The comparison of baseline characteristics and medication use between ChEI users and nonusers is listed in Table 1. Generally, when comparing ChEI users with nonusers, unmatched patients were substantially younger, more often women, and more likely to be patients with diabetes mellitus, hypertension, hyperlipidemia, chronic liver disease, or peripheral vascular disease; ChEI users were also more likely to use antiplatelet or anticoagulation medications, statins, beta-blockers, ACEIs, or ARBs but less likely to be diagnosed as having chronic kidney disease than nonusers were. No substantial differences between ChEI users and nonusers were observed for prior MI, prior stroke, chronic lung disease, atrial fibrillation, or venous thromboembolism before PSM. In the ChEI group, most patients used donepezil (72.5%), 16.2 and 11.3% of them used rivastigmine and galantamine, respectively.

**Table 1:** Baseline characteristics before and after propensity score matching.

	**Unmatched patients**			**Matched patients**		
	**Control**	**ChEIs**	***P*-value**	**Control**	**ChEIs**	***P*-value**
Patient number, *n*	6,712	699		1,336	668	
Female gender, *n* (%)	4,125 (61.5)	466 (66.7)	0.008	860 (64.4)	440 (65.9)	0.540
Age, years	77.8 ± 9.2	76.2 ± 8.5	<0.001	76.2 ± 9.1	76.3 ± 8.5	0.885
Diabetes mellitus, *n* (%)	1,925 (28.7)	245 (35.0)	0.001	431 (32.3)	231 (34.6)	0.322
Hypertension, *n* (%)	3,749 (55.9)	475 (68.0)	<0.001	903 (67.6)	445 (66.6)	0.699
Hyperlipidemia, *n* (%)	1,452 (21.6)	281 (40.2)	<0.001	493 (36.9)	251 (37.6)	0.806
Prior MI, *n* (%)	122 (1.8)	14 (2.0)	0.842	22 (1.6)	14 (2.1)	0.593
Prior stroke, *n* (%)	2,423 (36.1)	267 (38.2)	0.291	511 (38.2)	253 (37.9)	0.909
Chronic lung disease, *n* (%)	2,142 (31.9)	248 (35.5)	0.061	484 (36.2)	235 (35.2)	0.681
Chronic liver disease, *n* (%)	1,012 (15.1)	152 (21.7)	<0.001	270 (20.2)	141 (21.1)	0.681
Chronic kidney disease, *n* (%)	279 (4.2)	10 (1.4)	0.001	15 (1.1)	10 (1.5)	0.618
Atrial fibrillation, *n* (%)	185 (2.8)	16 (2.3)	0.548	24 (1.8)	15 (2.2)	0.607
Peripheral vascular disease, *n* (%)	610 (9.1)	100 (14.3)	<0.001	158 (11.8)	94 (14.1)	0.175
Venous thrombosis or embolism, *n* (%)	66 (1.0)	9 (1.3)	0.571	18 (1.3)	6 (0.9)	0.514
Antiplatelet, *n* (%)	811 (12.1)	165 (23.6)	<0.001	281 (21.0)	150 (22.5)	0.501
Anticoagulation, *n* (%)	19 (0.3)	7 (1.0)	0.009	8 (0.6)	3 (0.4)	0.761
Statin, *n* (%)	395 (5.9)	157 (22.5)	<0.001	240 (18.0)	127 (19.0)	0.610
Beta-blocker, *n* (%)	889 (13.2)	171 (24.5)	<0.001	160 (24.0)	304 (22.8)	0.550
ACEi/ARB, *n* (%)	911 (13.6)	234 (33.5)	<0.001	416 (31.4)	204 (30.5)	0.824
Type of ChEIs						
Donepezil, *n* (%)	0 (0.0)	507 (72.5)		0 (0.0)	494 (74.0)	
Rivastigmine, *n* (%)	0 (0.0)	113 (16.2)		0 (0.0)	101 (15.1)	
Galantamine, *n* (%)	0 (0.0)	79 (11.3)		0 (0.0)	73 (10.9)	

*ACEi, angiotensin-converting enzyme inhibitor; ARB, angiotensin receptor blocker; ChEIs, Cholinesterase inhibitors; MI, myocardial infarction*.

After 1:2 PSM, 668 patients (ChEI users) were included in the exposure group and 1,336 patients (ChEI nonusers) in the control group. All matched characteristics and medications listed on the left side of Table 1 were similar for the exposure and control groups. The types of ChEIs in the matched exposure group included donepezil 74.0%, rivastigmine 15.1%, and galantamine 10.9%.

### Clinical Outcomes

**Figure 2:**
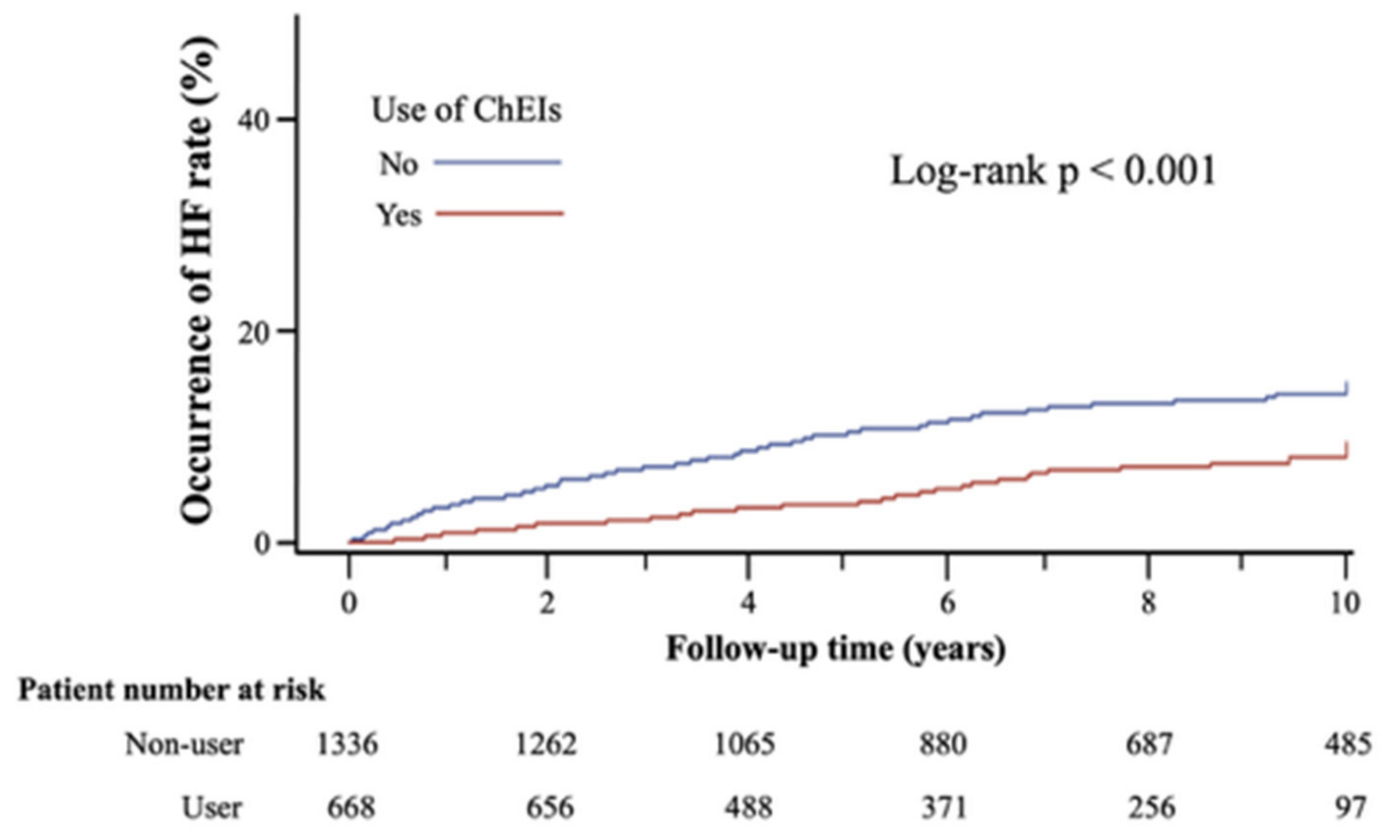
Kaplan-Meier survival curves of new onset of HF after 10-years follow-up in matched cohort.

The Kaplan–Meier survival curves for new-onset HF between ChEI users and controls (log-rank *p* < 0.001) are displayed in Figure 2. Table 2 lists the clinical outcomes and relative risks between ChEI users and controls after 10-year follow-up. New onset HF at 10-year follow-up was 6.1% for ChEI users and 13.0% for nonusers. The incidence of new-onset HF among donepezil, rivastigmine, and galantamine were 34/494 (6.88%), 1/101 (1%), 5/73 (6.85%), respectively and there were no statistical significance differences (*p*-value = 0.071). The incidence rate for new-onset HF per 1,000 patient-years was 9.5 for ChEI users and 18.1 for controls. Compared with nonusers, ChEI users had a lower risk of new onset HF both in the Cox proportional hazards model (HR 0.51; 95% CI: 0.36–0.72, *p* < 0.001) and in the competing risk regression analysis (HR 0.48; 95% CI: 0.34–0.68, *p* < 0.001).

**Table 2:** Clinical outcomes and relative risks between ChEI users and controls after 10-year follow-up.

**Clinical events in groups**	**Events, n (%)**	**Person-years**	**Incidence per 1,000 person-years**	**Cox proportional model**		**Competing risk regression**	
				**HR (95% CI)**	***P*-value**	**HR (95% CI)**	***P*-value**
**HF**							
Control	174 (13.0)	9,630	18.1	1.00 [Reference]	–	1.00 [Reference]	–
ChEIs	41 (6.1)	4,332	9.5	0.51 (0.36–0.72)	<0.001	0.48 (0.34–0.68)	<0.001
**MI**							
Control	22 (1.6)	10,483	2.1	1.00 [Reference]	–	1.00 [Reference]	–
ChEIs	12 (1.8)	4,424	2.7	1.20 (0.58–2.50)	0.620	1.09 (0.52–2.28)	0.821
**Cardiovascular death**							
Control	119 (8.9)	10,011	11.9	1.00 [Reference]	–	1.00 [Reference]	–
ChEIs	31 (4.6)	4,377	7.1	0.59 (0.40–0.88)	0.009	0.55 (0.37–0.82)	0.003

*ChEI, Cholinesterase inhibitor; CI, confidence interval; HF, heart failure; HR, hazard ratio; MI, myocardial infarction*.

After 10-year follow-up, cardiovascular death had occurred in 4.6% of patients in the ChEI user group and in 8.9% of patients in the nonuser group. The incidence rate of cardiovascular death per 1,000 patient-years was 7.1 for ChEI users and 11.9 for nonusers. Compared with controls, the use of ChEIs reduced the risk of cardiovascular death by 41% in the Cox proportional hazards model (HR 0.59; 95% CI: 0.40–0.88, *p* = 0.009) and by 45% in the competing risk regression analysis (HR 0.55; 95% CI: 0.37–0.82, *p* = 0.003).

MIs occurred in 1.8% of the patients in the ChEI group and in 1.6% of the patients in the nonuser group after 10-year follow-up. The incidence rates of MI per 1,000 patient-years were 2.7 for ChEI users and 2.1 for nonusers. The risk of MI in ChEI users was not significantly different from that in the control group in the Cox proportional hazards model (HR 1.20; 95% CI: 0.58–2.50, *p* = 0.620) or in the competing risk regression analysis (HR 1.09; 95% CI: 0.52–2.28, *p* = 0.821).

### Subgroups Analysis for Primary Preventive Effect ChEIs on New-Onset HF

**Figure 3:**
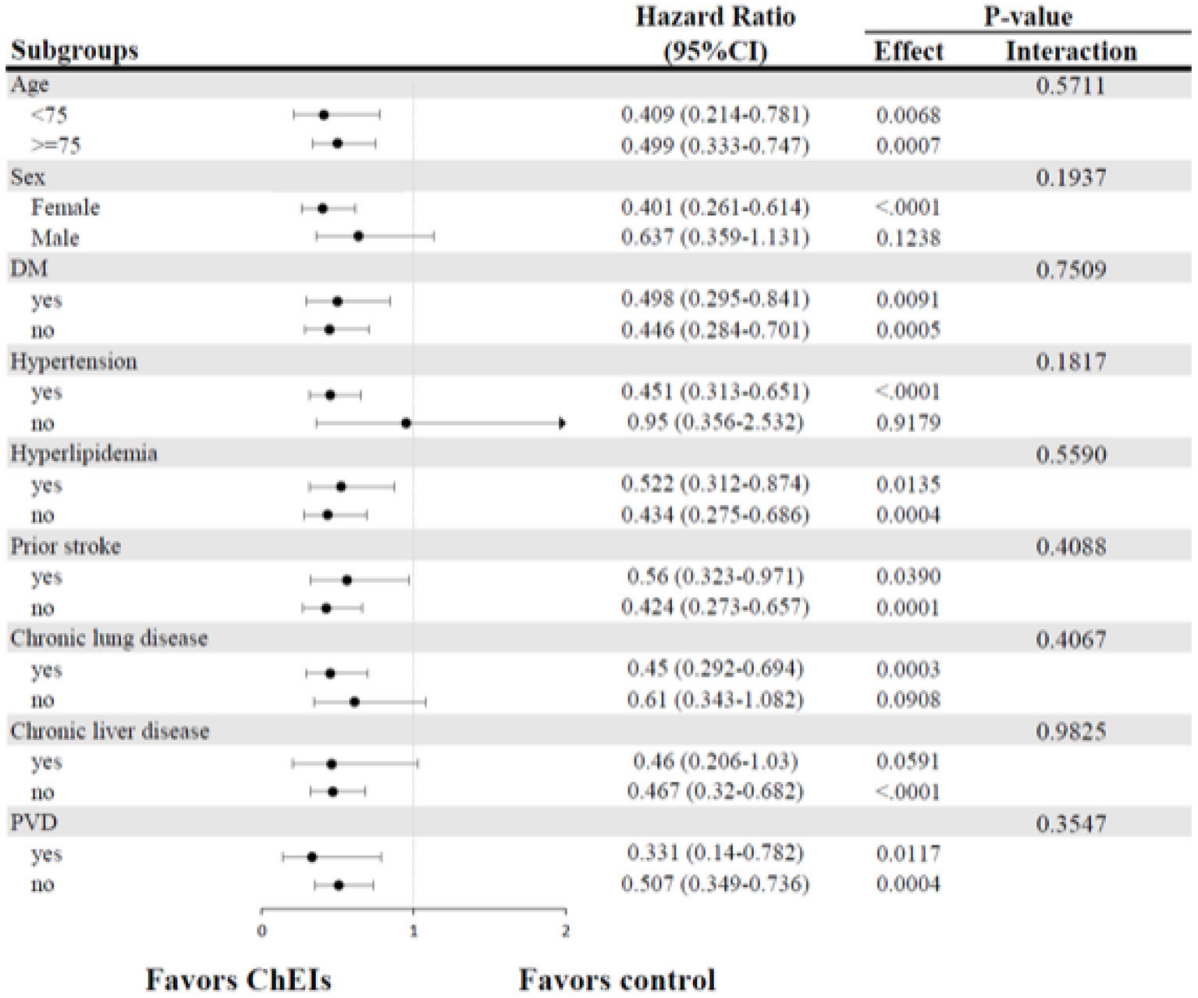
Forest plots of subgroup analysis for new-onset HF. CI, confidence intervals; DM, diabetes mellitus; HF, heart failure; PVD, peripheral vascular disease.

Figure 3 displays the results of subgroup analysis for the occurrence of HF according to baseline characteristics in the matched study populations. In comparisons with the control group, the preventive benefit of ChEIs was consistent across all exploratory subgroups without statistically significant treatment-by-subgroup interactions.

## Discussion

The cardioprotective effect of ChEIs has been noted during the past decade in animal and clinical studies. However, due to the inhibition of platelets activation, microphage production of cytokines, and reductions in oxidative stress, most of these studies have focused on the benefit of ChEIs in reducing the occurrence of atherosclerotic events (17, 20, 21). The main findings of the present study demonstrate that chronic use of ChEIs is associated with a lower incidence of new-onset HF and cardiovascular death, and this potential preventive effect is not related to the occurrence of MIs in patients with AD.

The incidences of HF and AD increase with age (1), but the incidence of new-onset HF in patients with AD has never been examined. In the general population, the incidence for HF (per 1,000 person-years) increases from <5 in patients 50–59 years of age to more than 25 in patients 80–89 years of age and more than 40 in those 90 years of age or older (22, 23). The incidence rate for AD (per 1,000 person-years) increases from 2.8 in patients 65–69 years age to 56 in patients 90 years of age or older (24). A long-term observational study reported that most elderly patients with HF had dementia and usually the onset of dementia had occurred before patients were diagnosed as having HF (25). Studies indicate that HF, with a prevalence rate of ~10%, is one of the most common comorbidities in patients with AD (26). The results of the present study are consistent with these findings in that the prevalence of HF in the total study cohort was 10.6% (6.1% in ChEI users vs. 13.0% in nonusers). In addition, this study is the first to report a significant reduction in new-onset HF in ChEI users compared with control; the incidence of new-onset HF was 18.1 per 1,000 person-years in control patients with AD at a mean age of 76 years; after using ChEIs for 10 years, the incidence of new-onset HF significantly decreased to 9.5 per 1,000 person-years.

The interplay between HF and AD is complex and has long been noted. HF and AD share similar common risk factors such as age, hypertension, diabetes mellitus, and obesity (27, 28). In addition, AD and HF share some genetic variants (29, 30). Low cardiac output in patients with HF leads to chronically inadequate cerebral blood flow and increased oxidative stress, which are followed by a series of pathological consequences such as protein and enzyme dysfunctions, amyloid beta protein deposition, and eventual neuronal cell death (31, 32). Therefore, HF is recognized as a risk factor in the development of AD (33). Conversely, AD patients are also a high-risk population for the development of cardiovascular diseases. Troncone et al. found that patients with AD had a thicker left ventricle than those without AD. The hallmark feature of AD, the accumulation of beta-amyloid proteins in the extracellular space of the brain, can also be found in the left ventricle (34). This amyloid deposition mechanism places patients with AD at a higher risk for the occurrence of atherosclerosis and HF (35). Because of involvement of cholinesterase in amyloid fibril formation process, using of ChEIs not only can reduce beta-amyloid deposition in the brain, but also can delay the development of HF through the same mechanism (36).

Autonomic imbalance, including sympathetic nervous activation and parasympathetic withdrawal, is another pathogenetic mechanism in the development and progression of HF (3, 4). Whether parasympathetic stimulation therapy is a treatment for HF is still under investigation. A small clinical trial demonstrated the benefit of cholinergic agents on exercise hemodynamic profile improvement in patients with HF (37). In addition, baroreflex activation appeared to be safe and effective for patients with HF (10). Despite sparseness of cholinergic innervation of ventricular myocardium, Non-neuronal cardiac cholinergic system plays an important role in cardiac homeostasis regulation (38, 39). Acetylcholine or muscarinic receptor agonist, through upregulation of choline acetyltransferase and choline transporter in cardiomyocytes, can synthesized acetylcholine themselves. This autocrine or paracrine effects can circumvent mitochondrial overshoot to protect cardiomyocytes from energy depletion when suffering from stress or energy demand (40, 41). Application of cholinergic agents to prevent HF development in large scale study has never been reported. Though there are theoretically cardiovascular benefits, intolerable side effects or toxicity limit the long-term application of these cholinergic agents (42, 43). The safety of the chronic use of ChEIs, which is well known in patients with AD, indicates that this class of medications has greater potential than other cholinergic agents do in the primary prevention of HF (44). *In vivo* and *vitro* studies proved donepezil, the most common used ChEIs, was different from other cholinergic agents in increasing expression of choline acetyltransferase promoter and activating intrinsic cardiac acetylcholine synthesis to positively regulate cardiomyocytes in terms of its energy metabolism, ischemic response, angiogenesis, and oxidative stress (40). This may be another reason to explain why ChEI is effective to prevent development of HF in this present study.

The strength of this study was the exclusion of patients with a diagnosis of HF to elucidate the possible primary preventive effect of using ChEIs. In addition, this study cohort also excluded patients with vascular dementia or Parkinson's disease and included a few numbers of patients with prior MI, with chronic kidney disease, and with atrial fibrillation, thus reducing the presence of comorbidity-related confounders in the evaluation of ChEI efficacy. Though animal studies have proved the effect of chronic parasympathetic nervous activation on the prevention of cardiac chamber remodeling and the development of HF (45), the present study is the first human study to report the potential primary preventive benefit of using a chronic parasympathetic modification agent–ChEI in association with a lower incidence of new-onset HF. Additional studies should examine whether this preventive benefit of ChEIs can extend to other populations without AD or whether ChEIs may be an effective treatment for patients with already established HF.

However, this study has several limitations. First, because of the retrospective nature of the study, the study groups may have had inherent differences. Although we used PSM to balance differences associated with major characteristics at baseline, hidden bias may still have occurred. Second, the participants in this study were all elderly patients with dementia. Patients with severe dementia may lose their sense of illness related to cardiovascular diseases. However, patients' cognitive condition, functional ability, and the effects of ChEIs will remain in this database. Third, this is a retrospectively database-based study, so the serum levels of acetylcholine and cholinesterase before and after ChEIs are not available to identify the response of ChEIs. Finally, an underestimation resulting from noncompliance is likely because information available about prescribed medications may not reflect their actual use.

In conclusion, this real-world observational study demonstrated that the use of ChEIs in patients with AD is associated with lower incidence of new-onset HF, which may translate to a lower risk of cardiovascular death, and this possible preventive effect is not related to MI.

## Data Availability Statement

The original contributions presented in the study are included in the article/Supplementary Material, further inquiries can be directed to the corresponding author.

## Ethics Statement

The Institutional Review Board of Chang Gung Memorial Hospital approved the protocol for this cohort study and waived the need for informed consent because all patient data were deidentified before analysis (IRB No. 202100758B1).

## Author Contributions

M-JH: conceptualization. M-JH, D-YC, and C-HL: methodology. C-LW, Y-JC, Y-TH, and S-HC: formal analysis and investigation. M-JH, S-HC, and Y-TH: writing review and editing. S-HC and Y-TH: funding acquisition and supervision. All authors contributed to the article and approved the submitted version.

## Funding

This work was supported by grants from Chang Gung Medical Research Program (grant number CMRPG3I0092).

## Conflict of Interest

The authors declare that the research was conducted in the absence of any commercial or financial relationships that could be construed as a potential conflict of interest.

## Publisher's Note

All claims expressed in this article are solely those of the authors and do not necessarily represent those of their affiliated organizations, or those of the publisher, the editors and the reviewers. Any product that may be evaluated in this article, or claim that may be made by its manufacturer, is not guaranteed or endorsed by the publisher.
