# Supplementary material for: Association Between Cholinesterase Inhibitors and New-Onset Heart Failure in Patients With Alzheimer's Disease: A Nationwide Propensity Score Matching Study
Source: Front Cardiovasc Med. 2022 Mar 16;9:831730. doi: 10.3389/fcvm.2022.831730 (PMC8966646; doi:10.3389/fcvm.2022.831730)
Supplement: Supplementary file 1 [file Table_1.DOCX]

**Supplement Table. Diagnosis codes of study covariates and outcomes**

|  | **ICD-9 diagnosis codes** | **ICD-10 diagnosis codes** |
| --- | --- | --- |
| **Covariates** |  |  |
| Diabetes mellitus | 250.x | Z13.1, 024.4, 024.92, E08.*, E09.*, E10.*, E11.*, E13.* |
| Hypertension | 401, 402 | I10, I11, I12, I13, I15 |
| Hyperlipidemia | 272.0, 272.1, 272.2, 272.3, 272.4 | E78.0, E78.1, E78.2, E78.3, E78.4, E78.5 |
| Prior MI | 410, 412 | I21.*, I21.*, I22.*, I23.*, I25.2 |
| Prior stroke | 430, 431, 432, 433, 434, 435, 436, 437, 438, 362.34 | I60.*, I61.*, I62.*, I63.*, I65.*, I66.*, I67.*, I68.*, I69.*, H34.0 |
| Chronic lung disease | 490, 491, 492, 493, 494, 495, 496, 497, 498, 499, 500, 501, 502, 503, 504, 505, 416.8, 416.9, 506.4, 508.1, 508.8 | J40, J41.*, J42.*, J43.*, J44.*, J45.*, J47.*, J60.*, J61.*, J62.*, J63.*, J64.*, J65.*, J66.*, J67.*, J68.*, J69.*, J70.* |
| Chronic liver disease | 570, 571, 572, 571.0, 571.1, 571.2, 571.3, 571.4, 571.5, 571.6, 571.8, 571.9, 571.40, 571.41, 571.49 | K72.*, K73.*, K74.*, K75.*, K76.*, K77.* |
| Chronic kidney disease | 580, 581, 582, 583, 584, 585, 586, 587, 588, 589 | N00.*, N01.*, N02.*, N03.*, N04.*, N05.*, N06.*, N07.*, N08.* |
| Atrial fibrillation | 427.31 | I48.* |
| Peripheral vascular disease | 440, 441, 093.0, 437.3, 443.1, 443.2, 443.3, 443.4, 443.5, 443.6, 443.7, 443.8, 443.9, 447.1, 557.1, V43.4 | I67.*, I70.*, I73.* |
| Venous thrombosis or embolism | 453.8, 415.1 | I82.*, I26.* |
| **Outcomes** |  |  |
| Heart failure | 428.*, 402.01, 402.11, 402.91, 404.01, 404.03, 404.11, 404.13, 404.91, 404.93, 785.51, 425.4, 425.5, 425.6, 425.7, 425.8, 425.9 | I50.1, I50.8*, I50.9, I11.0, I13.0, I13.2, R57.0 |
| Myocardial infarction | 410, 412 | I21.*, I21.*, I22.*, I23.*, I25.2 |
| Cardiovascular death | 362, 398, 402, 404, 410, 412, 425, 428, 430, 431, 432, 433, 434, 435, 436, 437, 438 | I21, I22, I25.2, I42, I43, I50, I60, I61, I62, I63, I65, I66, I67, I68, I69, G45, G46 |

ICD-9, International Classification of Diseases, Ninth Revision;
ICD-10, International Classification of Diseases, Tenth Revision
